# Supplementary material for: An open-source framework for large-scale, flexible evaluation of biomedical text mining systems
Source: J Biomed Discov Collab. 2008 Jan 29;3:1. doi: 10.1186/1747-5333-3-1 (PMC2276192; doi:10.1186/1747-5333-3-1)
Supplement: Additional file 1 — Evaluation platform source code. The additional file contains the software evaluation framework discussed in this paper. The most current release can be downloaded from SourceForge.net [1]. See the accompanying README file for instructions on installation and use. Also included in the distribution is a collection of UIMA wrappers for some commonly used BioNLP tools and annotated corpora. [file 1747-5333-3-1-S1.zip › bionlp_uima_repository-1.0.1/docs/api/src-html/edu/uchsc/ccp/util/nlp/annotation/Annotator.html]

```
001    /*
002     *
003     * The Original Code is UCHSC Utilities.
004     *
005     * The Initial Developer of the Original Code is University of Colorado.  
006     * Copyright (C) 2005.  All Rights Reserved.
007     *
008     * UCHSC Utilities was developed by the Center for Computational Pharmacology
009     * (http://compbio.uchsc.edu) at the University of Colorado Health 
010     *  Sciences Center School of Medicine with support from the National 
011     *  Library of Medicine.  
012     *
013     *
014     * Contributor(s):
015     *   William A. Baumgartner <william.baumgartner@uchsc.edu> (Original Author)
016     *   
017     * Created: January 2006
018     * Dependencies:
019     * TODO:   
020     * Changes:
021     * Notes: 
022     */
023    package edu.uchsc.ccp.util.nlp.annotation;
024    
025    import java.io.PrintStream;
026    
027    public class Annotator implements Comparable {
028    
029        private String firstName;
030    
031        private String lastName;
032    
033        private String affiliation;
034    
035        private Integer annotatorID;
036    
037        public Annotator(Integer annotatorID, String firstName, String lastName, String affiliation) {
038            this.annotatorID = annotatorID;
039            if (firstName != null) {
040                this.firstName = firstName;
041            } else {
042                this.firstName = "";
043            }
044            if (lastName != null) {
045                this.lastName = lastName;
046            } else {
047                this.lastName = "";
048            }
049            if (affiliation != null) {
050                this.affiliation = affiliation;
051            } else {
052                this.affiliation = "";
053            }
054        }
055    
056        public String getAffiliation() {
057            return affiliation;
058        }
059    
060        public void setAffiliation(String affiliation) {
061            if (affiliation != null) {
062                this.affiliation = affiliation;
063            } else {
064                this.affiliation = "";
065            }
066        }
067    
068        public Integer getAnnotatorID() {
069            return annotatorID;
070        }
071    
072        public void setAnnotatorID(Integer annotatorID) {
073            this.annotatorID = annotatorID;
074        }
075    
076        public String getFirstName() {
077            return firstName;
078        }
079    
080        public void setFirstName(String firstName) {
081            if (firstName != null) {
082                this.firstName = firstName;
083            } else {
084                this.firstName = "";
085            }
086        }
087    
088        public String getLastName() {
089            return lastName;
090        }
091    
092        public void setLastName(String lastName) {
093            if (lastName != null) {
094                this.lastName = lastName;
095            } else {
096                this.lastName = "";
097            }
098        }
099    
100        public String getStorageLine() {
101            return annotatorID + "|" + firstName + "|" + lastName + "|" + affiliation;
102        }
103        
104        public void printStorageLine(PrintStream ps) {
105            ps.println(getStorageLine());
106        }
107    
108        // compares on strings only. ID is not considered
109        public int compareTo(Object annotator) {
110            if (annotator instanceof Annotator) {
111                String firstName = ((Annotator) annotator).getFirstName();
112                String lastName = ((Annotator) annotator).getLastName();
113                String affiliation = ((Annotator) annotator).getAffiliation();
114    
115                if (firstName == null) {
116                    firstName = "";
117                }
118    
119                if (lastName == null) {
120                    lastName = "";
121                }
122    
123                if (affiliation == null) {
124                    affiliation = "";
125                }
126    
127                if (this.firstName.equalsIgnoreCase(firstName) & this.lastName.equalsIgnoreCase(lastName)
128                        & this.affiliation.equalsIgnoreCase(affiliation)) {
129                    return 0;
130                } else {
131                    return -1;
132                }
133            } else {
134                return -1;
135            }
136        }
137    
138        @Override
139        public boolean equals(Object annotator) {
140            if (compareTo(annotator) == 0) {
141                return true;
142            } else {
143                return false;
144            }
145        }
146    
147        @Override
148        public int hashCode() {
149            String hashkey = firstName + "|" + lastName + "|" + affiliation;
150            hashkey = hashkey.toLowerCase();
151            return hashkey.hashCode();
152        }
153    }
```
